# Causal association between triglycerides and cholesterol-lowering medication with non-rheumatic valve disease: A 2-sample Mendelian randomization study

**DOI:** 10.1097/MD.0000000000038971

**Published:** 2024-07-19

**Authors:** Kaiyuan Li, Xiaowen Wang, Peng Liu, Jun Ye, Li Zhu

**Affiliations:** a Graduate School of Dalian Medical University, Dalian Medical University, Dalian, P.R. China; b Nanjing University of Chinese Medicine, Nanjing, P.R. China; c Department of Cardiology, The Second Affiliated Hospital of Nanchang University, Nanchang, P.R. China; d Department of Cardiology, The Affiliated Taizhou People’s Hospital of Nanjing Medical University, Taizhou, P.R. China.

**Keywords:** causal effect, cholesterol-lowering drugs, Mendelian randomization, non-rheumatic valve disease, triglycerides

## Abstract

Previous studies have found a possible causal relationship between triglycerides and lipid-lowering drugs and valvular disease. The aim of this study was to explore the potential causal relationship between triglycerides and lipid-lowering drugs and valvular disease using Mendelian randomization (MR) analysis. Data sets associated with triglycerides (441,016 participants and 12,321,875 single nucleotide polymorphisms [SNPs]) and cholesterol-lowering drugs (209,638 participants and 9851,867 SNPs) were retrieved from the Genome-Wide Association Study (GWAS) database. A total of 297 and 49 SNPs significantly associated with triglycerides and cholesterol-lowering drugs, respectively (*P* < 5 × 10^−8^), were identified. Similarly, data sets for non-rheumatic valve diseases (NVDs) (361,194 participants and 10,080,950 SNPs) were obtained from the GWAS database. Inverse variance weighting was used as the primary method for calculating the odds ratio (OR) and 95% confidence intervals (CI). The MR-Egger, weighted median, and weighted mode analyses were also used to test the robustness of the main results. The MR-Egger intercept test and the MR-PRESSO test were used to evaluate horizontal pleiotropy. Inverse variance weighted (IVW) results showed that both triglyceride and cholesterol-lowering medication were positively associated with NVDs (OR = 1.001, 95% CI 1.000–1.0012, *P* = 0.006; OR = 1.007, 95% CI 1.003–1.010; *P* = 0.002). This study suggests that both triglyceride and cholesterol-lowering medications are positively associated with NVDs, suggesting that lowering triglyceride levels or the use of cholesterol-lowering medications may reduce the incidence of NVDs. However, larger samples are required for further validation.

## 1. Introduction

Valvular heart disease (VHD) is a common cardiovascular disease that jeopardizes the life and health of human beings, and severe valvular disease has a serious impact on the prognosis of patients.^[1]^ The incidence of VHD increases with age, and its etiology is varied. In developing countries, rheumatic heart valve disease is the most common cause, whereas in developed countries, Non-rheumatic valve diseases (NVDs) are the main cause, but the pathogenesis of NVDs has not been clearly understood.^[2,3]^ Aortic stenosis is by far one of the most common heart valve disorders, the incidence of other types of valve disease is relatively low, and all of these valves eventually accelerate heart failure.^[4]^ Therefore, early prevention and treatment of associated valve disease can slow the progression of heart failure and other diseases.^[5]^

Heart valve tissue is composed of 3 main components: valvular interstitial cells (VICs), valvular endothelial cells (VECs), and extracellular matrix.^[6,7]^ VECs cover the surface of heart valves in direct contact with the blood, and can maintain valve homeostasis by regulating processes such as valve permeability and inflammatory cell adhesion. It has been suggested that when VECs are exposed to external stimuli such as inflammatory cell infiltration, lipid deposition, and foam cell formation, they can penetrate into VICs and promote the differentiation of VICs into an osteoblastic phenotype, leading to valve calcification.^[8]^

Abnormal lipid metabolism is one of the major risk factors for atherosclerotic cardiovascular disease and therefore also influences the development of valve disease.^[9,10]^ When valves are damaged, lipids such as cholesterol and related lipoproteins, Lipoprotein (a), and oxidized high-density lipoprotein accumulate in the valve. When lipids infiltrate into the valve cells, the damaged VECs lead to the release of apoptotic vesicles, which subsequently trigger the secretion of inflammatory factors such as interleukin-1β and interleukin-6, ultimately damaging the valve.^[11]^

Most of the current research on triglyceride and cholesterol-lowering medication and the risk of NVDs is based on animal experiments and retrospective clinical studies.^[12–17]^ The bias caused by small sample sizes has resulted in a low level of evidence from these studies. Therefore, further studies are needed to clarify the relationship between them. Gene expression confers different characteristics in humans. Thousands of genetic variants have been reported for each allele in the population, and these variants are known as single nucleotide polymorphism (SNP).^[18]^ different genomes lead to variation in humans due to different gene expressions, the behavior of the population, the clinical manifestations of different diseases, and the response of the patients to the management protocols used.

This study performed a 2-sample Mendelian randomization (MR) analysis to elucidate the causal relationship between triglyceride and cholesterol-lowering medication and NVDs, and to provide new avenues for the prevention and treatment of NVDs in clinical practice.^[19,20]^ To our knowledge, this is the first study to use MR analysis to examine triglyceride and cholesterol-lowering medication with NVDs, and it provides a higher level of evidence than retrospective clinical studies.

## 2. Materials and methods

### 2.1. Study design

**Figure 1.**
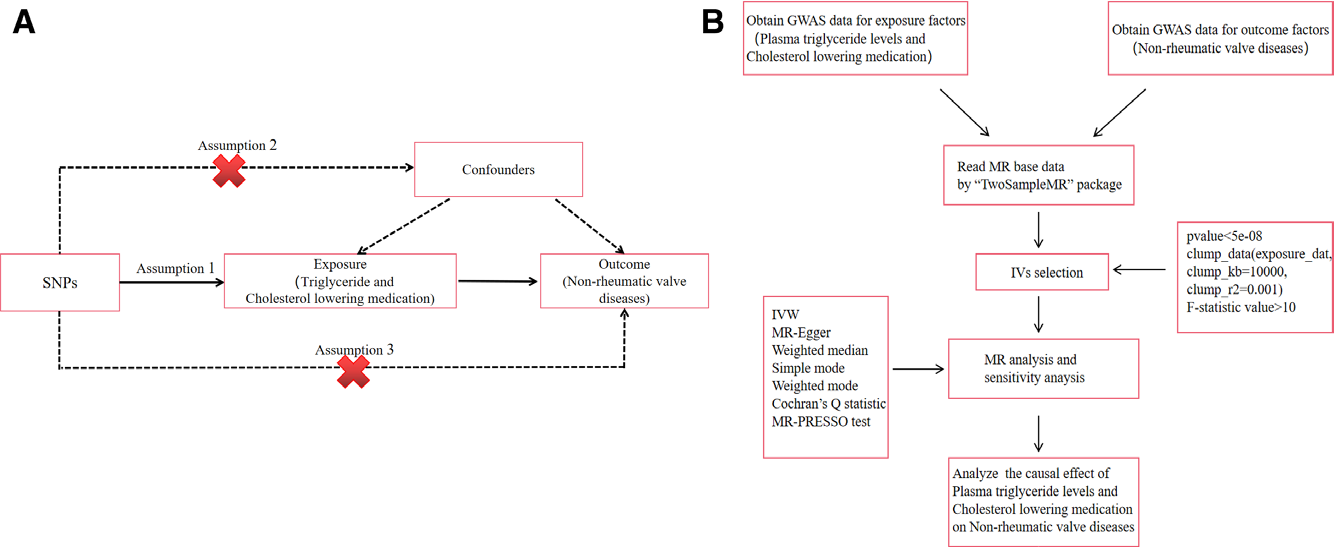
(A) Diagram for MR analysis; (B) Workflow of the study. MR is based on 3 hypotheses. First of all, SNPs identified as IVs should be strongly associated with exposure; secondly, selected SNPs must be independent of confounders; and finally, IVs are associated with NVDs (outcome) only triglycerides and cholesterol-lowering medication use (exposure), rather than through a direct association. MR = Mendelian randomization, NVDs = non-rheumatic valve diseases, SNPs = single nucleotide polymorphisms.

The specific process of this study is shown in Figure 1. The 2-sample MR applied in this study was based on genetic data obtained from the Global Genetic Alliance, which relies on 3 core assumptions: First, SNPs as instrumental variables (IVs) should be strongly associated with exposure; Second, the selected SNP must be independent of confounders; and finally IVs through triglyceride and cholesterol-lowering medication (exposure) was directly associated with NVDs (Fig. 1A).

### 2.2. Data sources

The datasets utilized in this MR study were sourced from the genome-wide association study (GWAS) database (https://gwas.mrcieu.ac.uk/). The dataset for triglycerides included 441,016 participants and 12,321,875 SNPs, contributed by the UK Biobank consortium with Richardson as the primary author. The cholesterol-lowering medication dataset comprised 48,450 treated participants and 161,188 controls, containing 9851,867 SNPs, with the MRC-i.e. consortium and Ben Elsworth as the primary author. The NVDs dataset involved 1606 patients and 359,588 controls, including 10,080,950 SNPs, contributed by the UK Biobank consortium with the Neale lab as the primary author. This study included European populations without distinguishing by age or gender. The datasets were obtained from published studies that had received ethical committee review, thus no further ethical approval was required. Additionally, as the datasets were compiled and analyzed by different authors at different times, there was no overlap between the 3 datasets (Table 1).

**Table 1:** Source of the GWAS data for triglycerides and cholesterol-lowering medication and non-rheumatic valve diseases.

Exposure/Outcome	Yr	Author	Participants	Number of SNPs	Web source if publicly
Triglycerides (ieu-b-111)	2020	Richardson, Tom (PMID:32203549)	441,016 individuals European ancestry	12,321,875	https://gwas.mrcieu.ac.uk/datasets/ieu-b-111/ (Access time: July 22, 2023)
Cholesterol-lowering medication (ukb-b-11740)	2018	Ben Elsworth	209,638 individuals (48,450 use cases and 161,188 controls) of European ancestry	9851,867	https://gwas.mrcieu.ac.uk/datasets/ukb-b-11740/ (Access time: July 22, 2023)
Non-rheumatic valve diseases (ukb-d-I9_NONRHEVALV)	2018	Neale lab	361,194 individuals (1606 cases and 359,588 controls) of European ancestry	10,080,950	https://gwas.mrcieu.ac.uk/datasets/ukb-d-I9_NONRHEVALV/ (Access time: July 22, 2023)

GWAS = Genome-wide association study, SNPs = single nucleotide polymorphisms.

### 2.3. IVs selection

To construct IVs for this study, SNPs from the datasets on triglycerides and cholesterol-lowering medications were extracted across the whole genome (*P* < 5 × 10^−8^), retaining those with a substantial physical distance (≥10,000 kilobases) and a lower likelihood of linkage disequilibrium (R^2^ < 0.001) (Fig. 1B). To mitigate bias caused by weak instrumental variables, each SNP in this study was required to have an F-statistic >10. The formula for calculating the F-statistic is: F= (β/SE)^2^.

### 2.4. Statistical analysis

The standard inverse variance weighting (IVW) method was used as the primary analysis method to calculate the strength of association between triglyceride and cholesterol-lowering medication and NVDs. The IVW method estimates the genetic locus-specific Wald ratio estimates by combining the genetically predicted behavioral phenotypes on the outcome, which assumes in its simplest form that all genetic variants are valid IVs, providing estimates and precision similar to 2-stage least squares. Wald ratios were calculated for each SNPs to estimate the causal effect of exposure on outcome. MR-Egger and weighted median were also performed as supplemental analyses.

### 2.5. Sensitivity analyses

Heterogeneity between SNP specificities was assessed using the Cochran Q test. If the heterogeneity was not statistically significant (*P* > 0.05), the fixed-effects model of IVW was used for MR analysis. Otherwise, the random effects model was used. The MR-Egger intercept test and the Mendelian randomized polytomous residuals and outliers (MR-PRESSO) test were used to detect polytomousness and correct for horizontal polytomousness by removing outliers. In addition, leave-one-out sensitivity analyses were performed to assess whether the MR results would be altered by a particular SNP.

## 3. Results

### 3.1. Genetic variant selection

**Figure 2.**
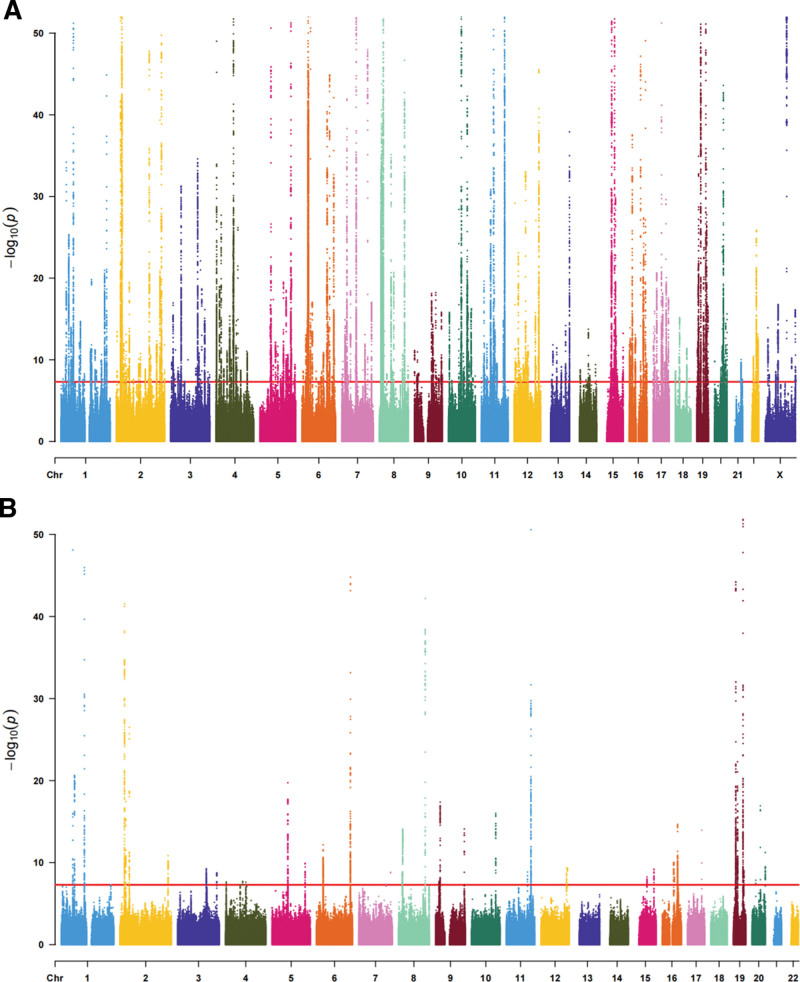
SNPs were obtained after screening according to the criteria. (A) and (B) 75,267 and 3734 significantly correlated SNPs (*P* < 5 × 10^−8^) were extracted from the GWAS datasets of triglyceride and cholesterol-lowering medication. GWAS = Genome-wide association study, SNPs = single nucleotide polymorphisms.

75,267 and 3734 significantly correlated SNPs (*P* < 5 × 10^−8^) were extracted from the GWAS datasets of triglyceride and cholesterol-lowering medication, respectively (Fig. 2A and B). Finally, by removing chain imbalance, removing weak variables and related complexities, 297 SNPs and 49 SNPs were finally selected as IVs for the 2 groups, respectively. This fulfills the assumption of strong correlation in MR studies. The detailed information of all SNPs is shown in Supplementary Table 1, http://links.lww.com/MD/N234 and 2, http://links.lww.com/MD/N235.

### 3.2. Causal effects of triglycerides on NVDs

**Figure 3.**
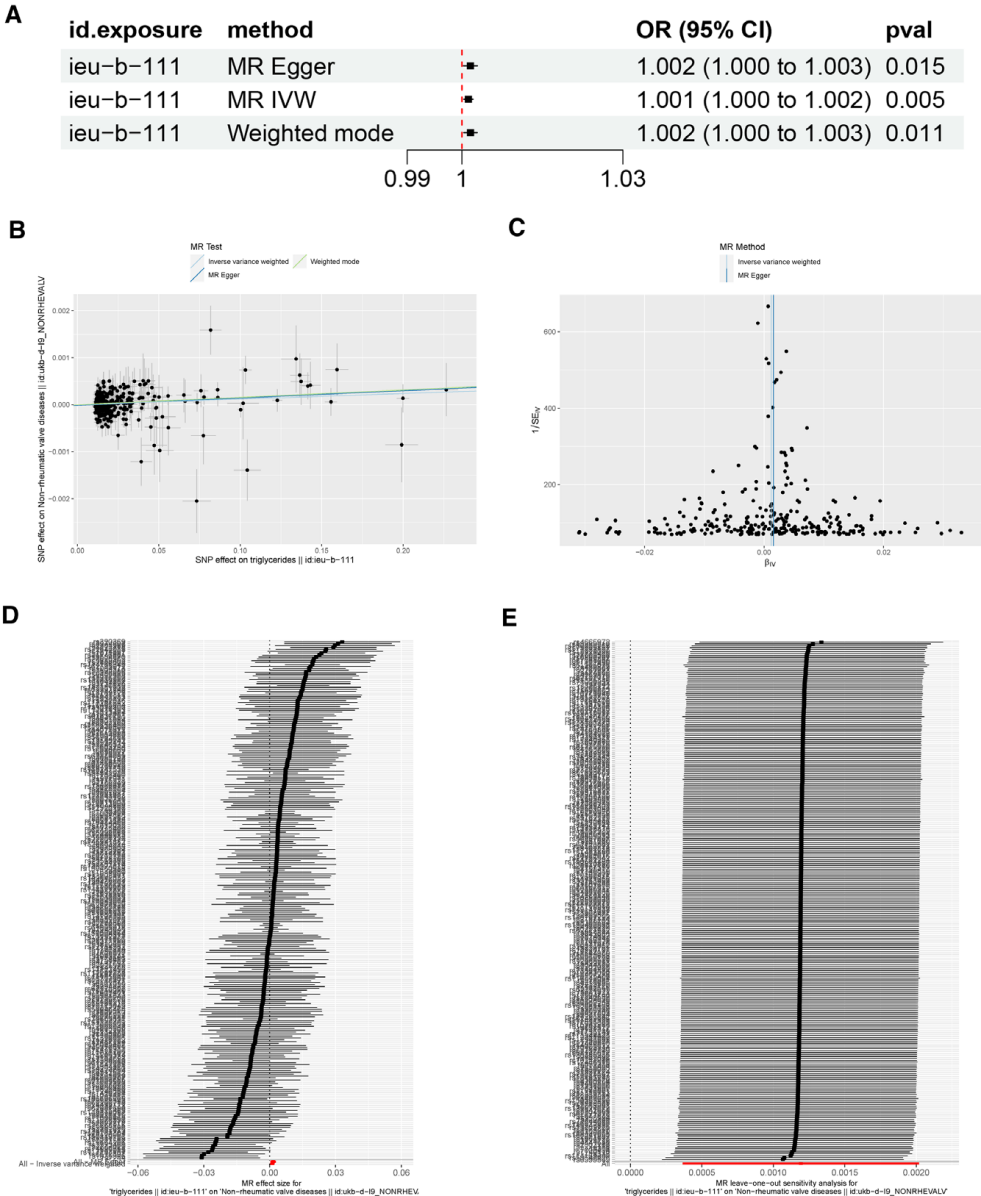
Causal effects of triglycerides on NVDs. (A) Statistical analyses of the causal relationship between triglycerides and NVDs; (B) Scatter plot of the effects of genetic variants on the triglycerides and NVDs. The slopes of the solid lines denote the magnitudes of the associations estimated from the MR analyses; (C) A Funnel plot of the causal effect of triglycerides and NVDs; (D) Fixed-effect IVW analysis of the causal association of triglycerides and NVDs; (E) Leave-one-out analysis plots for triglycerides and NVDs. IVW = inverse variance weighted, MR = Mendelian randomization, NVDs = non-rheumatic valve diseases.

The results of MR analyses of triglycerides and NVDs are shown in Figure 3A. The results of IVW analyses showed that triglycerides might be positively associated with the risk of developing NVDs (OR = 1.001, 95% CI 1.000–1.002, *P* = 0.005). Meanwhile, both MR-Egger (OR = 1.001, 95% CI 1.000–1.002, *P* = 0.015) and weight mode (OR = 1.001, 95% CI 1.000–1.002, *P* = 0.011) analyses also found that triglycerides may be positively associated with the risk of developing NVDs. No heterogeneity was found in the Cochran Q test (*P* = .056), and no horizontal multidimensionality was found in either the MR-Egger intercept test (intercept = −0.001, SE = −0.001, *P* = 0.445) or the MR-PRESSO test (*P* = 0.056) (Table 2). Leave-one-out analysis and Funnel plot both showed that no specific SNPs locus affected the overall estimate (Fig. 3B–E).

**Table 2:** Sensitivity analyses of the causal effect of triglyceride and cholesterol-lowering medication on non-rheumatic valve diseases.

Exposure	Directional horizontal pleiotropy			Cochran Q Test		MR-PRESSO
	egger_intercept	SE	*P* value	Q	Q_pval	
Triglyceride	−0.001	−0.001	0.445	308	0.056	0.075
Cholesterol-lowering medication	−0.001	0.001	0.393	61.3	0.065	0.068

MR = Mendelian randomization.

### 3.3. Causal effects of cholesterol-lowering medication treatment on NVDs

**Figure 4.**
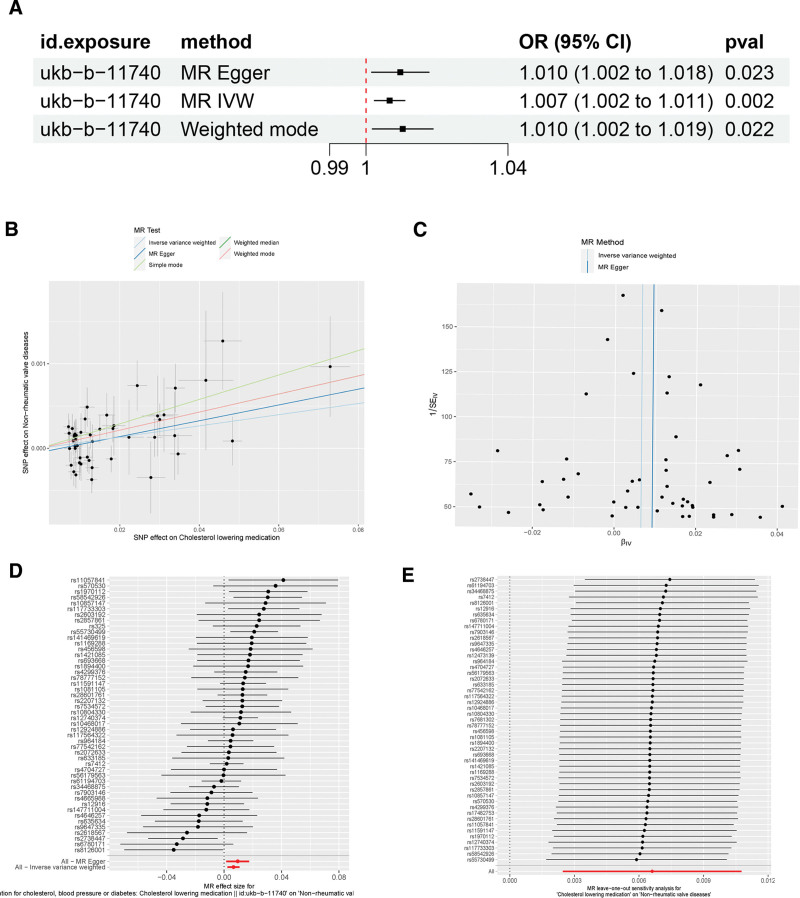
Causal effects of cholesterol-lowering medication on NVDs. (A) Statistical analyses of the causal relationship between cholesterol-lowering medication and NVDs; (B)Scatter plot of the effects of genetic variants on the cholesterol-lowering medication on NVDs; (C) A Funnel plot of the causal effect of cholesterol-lowering medication on NVDs; (D) Fixed-effect IVW analysis of the causal association of cholesterol-lowering medication on NVDs; (E) Leave-one-out analysis plots for cholesterol-lowering medication on NVDs. IVW = inverse variance weighted, NVDs = non-rheumatic valve diseases.

The results of MR analyses of cholesterol-lowering medications and NVDs are shown in Figure 4A. The results of IVW analyses showed that the use of cholesterol-lowering medications may slightly increase the risk of developing NVDs (OR = 1.007, 95% CI 1.003–1.010; *P* = 0.002). Meanwhile, MR-Egger (OR = 1.009, 95% CI 1.002–1.018; *P* = 0.023) and Weight Mode (OR = 1.010, 95% CI 1.002–1.019; *P* = 0.022), similarly demonstrated that the use of cholesterol-lowering medications might slightly increase the risk of developing NVDs no heterogeneity was detected by the Cochran Q test (*P* = 0.065) and the MR-Egger intercept test (intercept = −0.001, SE = 0.001, *P* = 0.393) and MR-PRESSO test (*P* = 0.068) found no evidence of horizontal multidimensionality (Table 2). Leave-one-out analysis and Funnel plot both showed that no specific SNPs locus affected the overall estimate (Fig. 4B–E).

## 4. Discussion

Previous observational clinical studies have found that abnormalities in lipid metabolism may play an important role in the pathogenesis of valvular heart disease, while triglycerides have been shown to be strongly associated with the risk of aortic stenosis, but correlation studies with other valvular diseases are still lacking.^[10,21,22]^ The present study identifies a potential causal relationship between triglycerides and NVDs by MR studies and provides evidence for continued exploration of the complex mechanisms of action between lipids and valvular disease.

Atherosclerosis is a chronic inflammatory pathological process that predisposes the vascular wall and is primarily characterized by lipid deposition and inflammatory cell infiltration.^[23,24]^ In a similar pathological setting, VECs cover the surface of heart valves and can be similarly affected by lipid metabolism, accelerating valve damage with lipid deposition and inflammatory cell infiltration.^[25,26]^ In previous fundamental experiments, It has been found that hyperlipidemia can lead to elevated levels of TGF-β1 and activate its downstream signaling pathways.^[27]^ At the same time, lipids can exacerbate valve calcification by inducing granulocyte alterations via the classical (p-Samd2/3) or nonclassical (p-ERK1/2) pathways, which can trigger an inflammatory response.^[28]^ In addition to this, lipoprotein (a) has been previously demonstrated in animal studies to promote the development of aortic valve calcification, which provides a pathophysiologic rationale for the use of cholesterol-lowering drugs to reduce the incidence of cardiovascular disease.^[29]^ The present MR study further confirms these findings by providing genetic evidence to support a possible potential causal relationship between triglycerides and NVDs.

This study sourced datasets on triglycerides and cholesterol-lowering medications from GWAS, employing standard criteria to select SNPs as IVs for exploring the effects of triglycerides and cholesterol-lowering medications on the risk of developing NVDs. The findings indicate that elevated levels of triglycerides increase the risk of cardiovascular diseases. Interestingly, the use of cholesterol-lowering medications was also associated with an increased risk of cardiovascular diseases. While lipid-lowering drugs, particularly statins, are beneficial in reducing the incidence of atherosclerotic cardiovascular diseases, their impact on valve diseases remains unclear. Some studies suggest potential benefits in slowing the progression of valve calcification, yet others have not demonstrated significant direct effects.^[30,31]^ Hence, the results of this study might highlight the complexity of valve disease pathology and the need for more targeted interventions. Additionally, due to the unavailability of the original article for the cholesterol-lowering medication dataset, it is unclear which specific lipid-lowering medications were included. However, the sensitivity analyses conducted in this study indicate a high degree of stability and reliability of the findings, thereby eliminating concerns regarding dataset overlap.

The current study has several significant strengths. First, the present study is based on large-scale GWAS summary statistics using MR analysis methods that are not susceptible to confounding factors. Second, chained unbalanced correlation analyses were performed for each instrumental variable, and potential weak instrumental bias was avoided by removing both the weak variables of interest and confounders.

This study also presents several limitations. Firstly, regarding population generalizability: the participants in this study were all of European descent, which may limit the applicability of the findings to other racial and ethnic groups. Future research should include more diverse populations to enhance the universality of the study results. Secondly, in terms of data sources and medication specificity: the datasets were sourced from GWAS databases, and the specific types of cholesterol-lowering medications included were not clarified. The lack of specificity regarding medication types may impact the interpretation of the study findings. Identifying which cholesterol-lowering medications have the most significant effect on cardiovascular diseases could refine treatment approaches. Lastly, while MR provides a more reliable estimate of causality than traditional observational studies, it cannot fully replicate the conditions of a randomized controlled trial. Therefore, the possibility of residual confounding or bias cannot be entirely ruled out. Expanding the scope of research to different populations, elucidating mechanisms of action, and conducting clinical trials will therefore be critical steps in applying these findings to clinical practice.

## 5. Conclusion

The study conducted an MR analysis to explore the causal links between triglycerides, cholesterol-lowering medication and NVDs, using comprehensive GWAS datasets. The findings indicate a positive association between both triglycerides and cholesterol-lowering medication with the incidence of NVDs, suggesting potential avenues for prevention and treatment through managing triglyceride levels and the use of cholesterol-lowering drugs. However, the study emphasizes the need for larger sample sizes to validate these results further.

## Acknowledgments

This research has been conducted using the UK Biobank Resource. We thank the UK Biobank for approving this research.

## Author contributions

**Conceptualization:** Kaiyuan Li, Xiaowen Wang, Peng Liu, Li Zhu.

**Data curation:** Kaiyuan Li, Peng Liu, Jun Ye.

**Formal analysis:** Kaiyuan Li, Xiaowen Wang, Peng Liu.

**Funding acquisition:** Jun Ye, Li Zhu.

**Investigation:** Kaiyuan Li, Peng Liu.

**Methodology:** Kaiyuan Li, Peng Liu.

**Project administration:** Kaiyuan Li, Peng Liu.

**Resources:** Kaiyuan Li, Peng Liu.

**Software:** Kaiyuan Li, Peng Liu.

**Supervision:** Kaiyuan Li, Jun Ye.

**Validation:** Peng Liu, Jun Ye.

**Visualization:** Kaiyuan Li, Xiaowen Wang, Peng Liu.

**Writing – original draft:** Kaiyuan Li, Xiaowen Wang, Peng Liu, Li Zhu.

**Writing – review & editing:** Kaiyuan Li, Peng Liu, Jun Ye, Li Zhu.

## Supplementary Material




