# Supplementary material for: Causal association between triglycerides and cholesterol-lowering medication with non-rheumatic valve disease: A 2-sample Mendelian randomization study
Source: Medicine (Baltimore). 2024 Jul 19;103(29):e38971. doi: 10.1097/MD.0000000000038971 (PMC11398802; doi:10.1097/MD.0000000000038971)
Supplement: Supplementary file 1 [file medi-103-e38971-s001.pdf]

SNP	beta.exposure	beta.outcome	eaf.exposure	eaf.outcome
rs1009360	-0.0184832	0.000104222	0.41859	0.415257
rs1015247	-0.0135209	1.77E-07	0.388656	0.387027
rs1017254	-0.0112291	0.000453552	0.408707	0.410215
rs1021097	0.0232557	-6.31E-05	0.131448	0.128996
rs1027758	-0.0173714	0.000423323	0.118591	0.118835
rs1037117	0.0172213	-0.000211039	0.254798	0.253882
rs1040594	-0.013209	-0.000167534	0.483077	0.484126
rs1043897	-0.014664	-0.000227423	0.415585	0.41518
rs1044808	-0.0247492	-0.000123378	0.081237	0.0823726
rs1045241	-0.0206988	2.50E-05	0.270943	0.271236
rs1051368	0.0247828	0.000102464	0.097403	0.0962765
rs1075076	0.0193855	-5.64E-05	0.709959	0.710805
rs1077300	-0.0149294	-7.75E-05	0.332276	0.332009
rs1077304	-0.0290398	0.000104735	0.39535	0.395333
rs1077540	0.0206571	7.79E-05	0.759694	0.761255
rs1077835	0.0473668	0.000179022	0.220459	0.21968
rs1079711	0.0157113	0.000120259	0.536622	0.536297
rs1081166	-0.0154098	-8.08E-05	0.173212	0.173515
rs1088302	-0.0144604	-2.04E-05	0.521569	0.52015
rs1089949	-0.0170082	0.000197533	0.161369	0.158341
rs1100046	-0.0148544	0.000233648	0.255269	0.254097
rs1103010	0.0160418	-0.000127574	0.260757	0.264242
rs1107859	0.0191708	-0.000310651	0.186613	0.18631
rs1111831	0.0193178	3.20E-05	0.593476	0.592476
rs1112245	-0.0481745	7.39E-05	0.611733	0.613093
rs1115005	-0.0419011	0.000138965	0.056946	0.0551899
rs1117196	0.0263393	0.000377836	0.060694	0.0588379
rs1118554	-0.0126395	2.58E-05	0.727914	0.727396
rs1118701	-0.0116437	0.000305888	0.550621	0.54998
rs1120637	0.0250232	-0.000206204	0.224815	0.224841
rs1122592	0.139849	0.000103058	0.028291	0.0288311
rs1123819	0.0231649	0.000347399	0.06731	0.0681323
rs1124035	0.0135651	-0.000159739	0.394026	0.394501
rs1133400	0.0137462	9.76E-05	0.219923	0.222076
rs1133444	0.0434468	0.000239273	0.060177	0.0610309
rs1141653	0.0818126	0.00158908	0.023157	0.0234674
rs1163768	0.0125866	3.28E-05	0.276291	0.277198
rs1166410	-0.0125831	0.000311725	0.374102	0.373482
rs1168430	-0.226505	-0.000320486	0.019327	0.0195795
rs1172331	-0.0732317	0.00205	0.015176	0.015367
rs1172872	-0.0394324	7.30E-05	0.028185	0.0286407
rs1172912	0.0297851	0.000378574	0.036536	0.0371532
rs1187823	-0.012703	3.79E-05	0.593373	0.595619
rs1190465	0.0410641	0.00042676	0.020999	0.0201757
rs1198045	0.0123903	-0.000104321	0.291468	0.293304
rs1218524	0.0175265	0.000176704	0.454836	0.456785
rs1242405	0.0191212	-0.000178511	0.232437	0.234326
rs1244080	0.0161	0.00017702	0.255149	0.253846
rs1244651	-0.0334207	-0.000122879	0.322889	0.324435
rs1247533	-0.0140007	0.000109245	0.260988	0.260253

rs1250474	-0.0152674	-0.000144485	0.192857	0.19343
rs1253067	-0.0121299	-2.41E-05	0.48466	0.48716
rs1254609	0.0278972	0.000278512	0.252369	0.250449
rs1288034	0.0209696	2.33E-05	0.159023	0.158274
rs1290204	-0.0129253	7.10E-05	0.313907	0.312708
rs1292065	-0.0139237	0.000170732	0.709244	0.710567
rs1292610	0.0126364	6.00E-05	0.454903	0.459357
rs1292809	-0.0282116	0.000305302	0.296442	0.296127
rs1306679	-0.0224583	0.000110784	0.090095	0.0902202
rs1310732	0.0300749	3.22E-05	0.074905	0.0748227
rs1310821	-0.0305169	9.67E-05	0.614859	0.61824
rs1311847	0.0149897	-3.22E-05	0.392654	0.389745
rs1316753	-0.0145732	-0.000235602	0.394062	0.393815
rs1326430	0.0189102	-0.000346432	0.151023	0.150624
rs1326597	0.0213535	0.000243824	0.082718	0.0823962
rs1326972	0.0349878	0.000497664	0.078416	0.0795949
rs1335432	-0.0154305	0.000136116	0.409787	0.413553
rs1338921	-0.0376242	0.000321745	0.392551	0.393259
rs1340819	-0.0121655	1.13E-05	0.345011	0.347139
rs134551	-0.0116536	-0.000118817	0.335438	0.337818
rs1347188	0.0139021	-0.000336656	0.245797	0.245158
rs1381917	-0.0471717	0.000863899	0.017021	0.0171344
rs1384220	-0.0271787	0.000414073	0.087231	0.087195
rs1393869	-0.0216903	-0.00034602	0.092129	0.0931125
rs1399746	0.143073	0.000414789	0.026131	0.0249577
rs1401072	-0.0227003	-4.52E-05	0.154707	0.15637
rs140288	-0.0132623	-1.17E-05	0.566711	0.56887
rs1420384	-0.01279	0.000153688	0.66699	0.667494
rs1430764	0.0403999	0.000508956	0.018647	0.0187268
rs1488277	0.0465516	8.75E-05	0.022544	0.0227799
rs1491428	0.0169358	4.00E-05	0.156359	0.15628
rs1497780	-0.0157568	0.000226255	0.32832	0.330214
rs1504191	-0.0505491	0.000975237	0.015259	0.0152609
rs1504605	0.0326952	9.40E-05	0.044425	0.04425
rs1505554	-0.0384234	-0.000425394	0.056868	0.0574747
rs1505644	-0.101751	-3.22E-05	0.011556	0.0114034
rs1512354	0.0521833	-0.000255137	0.015827	0.0160537
rs1534696	-0.0197664	-9.82E-05	0.541122	0.539587
rs1544980	0.0238704	6.71E-05	0.198644	0.199796
rs1567353	0.0148202	-5.70E-05	0.307644	0.308011
rs1718438	-0.0219251	1.41E-05	0.425367	0.426761
rs1732665	0.0174394	-0.000114051	0.238472	0.238686
rs174566	0.0485019	-6.22E-05	0.34974	0.350726
rs1753607	-0.0115701	-0.000228225	0.380342	0.377809
rs1758588	-0.0285849	0.00019609	0.591074	0.590435
rs1760801	-0.0202981	0.000129204	0.295768	0.298196
rs1799831	0.024506	0.000173855	0.156029	0.156514
rs1801689	-0.06613	-7.37E-05	0.030543	0.0298898
rs1835346	-0.0391877	0.00121057	0.023874	0.0241536
rs1866962	-0.104318	0.00139275	0.014693	0.0149094
rs1882475	-0.134286	-0.000975036	0.013117	0.01331

rs192249	-0.0137279	-0.000218106	0.651188	0.652352
rs193735	0.0329807	-0.000145761	0.036665	0.0371297
rs1938566	-0.0212127	-0.000268445	0.834537	0.833685
rs1999049(	-0.0118448	-0.000149794	0.320204	0.318874
rs2043085	-0.0308028	-5.03E-05	0.612184	0.612183
rs2068888	-0.0318216	-0.000144503	0.450732	0.44869
rs2070341	0.0113012	6.05E-05	0.60309	0.6031
rs2071887	0.016312	0.000384701	0.344804	0.343256
rs2081194	-0.0214115	-9.62E-05	0.600934	0.599653
rs2081687	-0.0261379	-0.000146355	0.663201	0.663427
rs2092203	0.0136756	0.000121009	0.481154	0.48089
rs2130382	-0.0251437	-3.61E-05	0.313212	0.31162
rs2131311	-0.0124181	-0.000176139	0.714636	0.714473
rs2131919	0.0173511	7.80E-05	0.164266	0.16324
rs213494	0.0156106	0.00019384	0.648345	0.64951
rs2137557	0.0117624	-1.54E-05	0.645612	0.643642
rs2187114	-0.0185151	-5.46E-05	0.101024	0.10241
rs2229092	0.0256807	0.000555194	0.062054	0.0625356
rs2237029	-0.0139399	0.000359155	0.601353	0.602895
rs2240466	-0.122769	-9.40E-05	0.122916	0.123789
rs2240533	-0.0129897	-0.000125444	0.309898	0.308364
rs2244278	-0.0269912	-7.97E-05	0.120553	0.120304
rs2267373	0.0215638	-0.000231379	0.580693	0.581313
rs2275852	0.025198	-0.000364392	0.052332	0.0506728
rs2276853	0.0111258	0.000163131	0.59607	0.597802
rs2302263	0.0436112	0.000505671	0.088863	0.0887243
rs2304969	-0.0161469	-0.000479267	0.144932	0.144623
rs2305746	0.0282544	0.000234725	0.933319	0.933622
rs2382825	-0.0134774	0.000243243	0.622954	0.623367
rs2407278	-0.0339449	-0.000246262	0.030154	0.0299755
rs2487294	0.0183191	-0.000120976	0.723477	0.725432
rs2519093	-0.021158	-0.000222337	0.184548	0.184254
rs2691553	0.0140801	0.000130843	0.51063	0.508568
rs2702544	0.0116368	-7.56E-05	0.658163	0.659517
rs275184	-0.0173487	6.92E-05	0.162051	0.161109
rs2773469	-0.0188717	0.000469622	0.733286	0.735477
rs278981	0.0125871	0.000113953	0.758155	0.757428
rs2811964	0.0196101	0.000375318	0.906954	0.906692
rs2812208	-0.048426	0.00028641	0.02089	0.0211404
rs2838331	0.0379005	0.000132688	0.624109	0.624272
rs2843911	0.0126014	-0.000175174	0.255528	0.256659
rs2857718(	-0.0163428	2.71E-06	0.664571	0.665096
rs2875292	0.0265556	-2.10E-05	0.445662	0.440558
rs2925979	-0.0322092	4.27E-05	0.699976	0.698975
rs2937124	-0.0182561	-0.000183036	0.362769	0.362185
rs2943645	0.0402918	2.49E-05	0.646594	0.646398
rs2983896	0.0137912	-0.000245819	0.21474	0.214054
rs308	-0.159413	-0.000749004	0.020597	0.0207505
rs3103310	0.0202549	0.00036398	0.241785	0.242223
rs320369	-0.0125327	-0.000413985	0.683292	0.681366
rs325485	-0.0117435	0.000166582	0.602889	0.602881

rs326222	0.0252203	-0.000166306	0.697957	0.696574
rs343	-0.141493	-0.000399296	0.083129	0.0825385
rs34345377	0.0138837	0.000242212	0.194496	0.19336
rs34682685	0.0337428	4.14E-05	0.104198	0.105191
rs35763453	0.02845	2.23E-05	0.058647	0.059141
rs36043408	-0.0128072	9.60E-05	0.502153	0.500599
rs36047579	0.0259717	0.000323757	0.114563	0.11474
rs3731696	0.0218939	0.000336927	0.121188	0.121527
rs37538	-0.0143974	-6.14E-05	0.601553	0.59915
rs3758413	0.0111232	-1.45E-05	0.417724	0.413943
rs3775228	0.0338197	0.000233849	0.399717	0.399816
rs3814883	0.0148926	4.98E-05	0.482498	0.484652
rs38189	-0.0153164	-9.54E-05	0.634401	0.633989
rs3820897	0.0197269	-6.02E-06	0.819861	0.820397
rs3936511	0.0461274	0.000370637	0.19191	0.191706
rs394872	0.0111807	-2.77E-05	0.536237	0.537083
rs3974807	0.0159776	0.000497573	0.189267	0.189962
rs4128205	0.0115011	3.65E-05	0.509173	0.512079
rs4134963	-0.0189544	0.000186065	0.189878	0.190585
rs41785	-0.0150445	2.21E-05	0.417487	0.416495
rs4253750	0.0177576	-0.000160738	0.214455	0.214695
rs4382584	0.0129432	-0.000224129	0.274334	0.275911
rs4471666	-0.022461	3.12E-06	0.069488	0.0684232
rs45445495	0.0289415	2.44E-05	0.049449	0.0498088
rs4662414	-0.0120574	0.000531158	0.446608	0.446577
rs4665972	-0.10025	0.000104967	0.604587	0.606977
rs4675812	-0.0142305	-0.000155801	0.587882	0.58754
rs4731701	-0.0325803	4.27E-05	0.493088	0.495723
rs4760254	-0.0281414	-0.000165573	0.23908	0.241714
rs4761234	-0.0140236	1.24E-05	0.484365	0.486708
rs4804101	0.0193079	0.000156609	0.439008	0.44138
rs480823	0.15572	5.97E-05	0.078996	0.0785367
rs483082	0.0862119	0.000151855	0.23535	0.237253
rs4841580	-0.0245383	-0.000292676	0.434941	0.434254
rs4962112	-0.0110418	-1.20E-05	0.528394	0.526751
rs4969179	-0.0177529	0.000294669	0.60445	0.603931
rs4976033	0.0177909	4.18E-05	0.402122	0.400954
rs499293	-0.0117912	-8.02E-05	0.65807	0.656488
rs53524119	0.0210699	-0.000166377	0.21449	0.213936
rs55646464	0.0121913	-4.57E-05	0.300087	0.301135
rs55767272	-0.0282011	9.16E-05	0.065077	0.0657122
rs55966194	-0.0178509	-6.53E-05	0.281997	0.284001
rs56397607	0.0174851	-3.70E-05	0.182538	0.182477
rs56902258	-0.0151434	-0.000154811	0.196198	0.197521
rs581080	0.0175432	-3.57E-05	0.819008	0.820131
rs58542926	-0.103184	-0.000740854	0.074608	0.0754304
rs6028716	-0.0128217	0.00018691	0.258428	0.259484
rs6073958	0.0556952	0.000189021	0.198674	0.198583
rs60856912	0.0248329	2.77E-05	0.162906	0.162603
rs61729990	-0.0558488	0.000490263	0.017888	0.0180762
rs61780049	0.0153212	0.000285517	0.14886	0.148067

rs6178548	0.0111206	3.65E-06	0.411762	0.410898
rs6183029	0.0287484	0.000349155	0.095791	0.0970863
rs6188596	-0.0313935	0.000114035	0.054205	0.0553421
rs6190507	0.199898	0.000135936	0.073841	0.0735082
rs6199368	-0.0234087	0.000244831	0.076413	0.0779069
rs6210271	0.0202348	-0.000272467	0.286196	0.286035
rs6212880	-0.0158746	4.08E-05	0.183216	0.183717
rs6227137	0.0419639	0.000451709	0.059967	0.0600023
rs6227409	0.0120882	0.000108179	0.424205	0.425817
rs6239724	0.0149941	0.000224751	0.22236	0.221294
rs6242798	-0.0133153	-4.99E-05	0.32217	0.324785
rs6245909	-0.0319865	-0.000254915	0.061781	0.0619784
rs6247352	-0.0211003	-2.78E-05	0.077998	0.0778506
rs6432622	-0.0108868	0.000168418	0.490264	0.488509
rs6506033	-0.0228948	-3.64E-05	0.072523	0.0715335
rs6517522	-0.0129031	0.000122757	0.498138	0.499016
rs6532798	0.013792	7.41E-05	0.697405	0.697549
rs6562773	-0.0120538	3.29E-05	0.547533	0.547315
rs6572807	0.0124759	-0.000373531	0.267065	0.267904
rs6700266	-0.0124503	4.63E-05	0.342465	0.341815
rs676210	-0.0735169	-5.09E-05	0.205293	0.204191
rs6792725	-0.0152543	-4.95E-05	0.692404	0.693357
rs6798169	0.0299225	-0.000421954	0.129887	0.128876
rs6798755	-0.0251021	0.000336961	0.065529	0.0659883
rs6800707	0.0298848	-0.000312097	0.810543	0.809543
rs6805924	0.0109684	-4.32E-05	0.430548	0.431785
rs6816767	-0.0160336	-3.10E-05	0.226252	0.224209
rs684773	0.0291133	-5.28E-05	0.766797	0.766965
rs6882076	0.0331011	2.63E-05	0.634285	0.635787
rs6924805	-0.0111556	6.45E-05	0.587637	0.589217
rs696825	-0.0202838	0.000231977	0.252926	0.251094
rs698927	-0.0183059	-0.000166136	0.183785	0.184105
rs6999569	-0.0860885	-0.000320495	0.470737	0.473234
rs7000494	0.136538	0.000628577	0.030042	0.0299258
rs7077812	0.0142284	-0.000237003	0.194911	0.192947
rs7134375	-0.0171435	0.000104618	0.431174	0.430691
rs7135509	-0.0121338	1.16E-05	0.293196	0.294131
rs7140110	0.0283172	0.000430966	0.298338	0.296958
rs7153812	0.0176494	0.00023524	0.122052	0.123019
rs7160340	0.0264991	-7.21E-05	0.136804	0.13516
rs7215055	0.0389233	0.000151246	0.062892	0.0623391
rs7239575	-0.0160767	4.68E-05	0.490494	0.493676
rs7244	0.0151978	-0.000470961	0.174166	0.17345
rs7255538	0.0653776	0.000204739	0.048975	0.0487735
rs7260374	-0.0125232	-0.00012017	0.315623	0.313635
rs7269163	-0.0154258	-0.00041753	0.187053	0.18724
rs7274718	0.0159491	0.000462848	0.598516	0.599104
rs7280147	-0.0307938	-0.000112046	0.091843	0.0926317
rs729761	0.0177868	6.02E-05	0.711859	0.71132
rs7302556	0.0138633	0.000279408	0.245879	0.245392
rs7308584	0.0149992	5.08E-05	0.184436	0.184796

rs73243877	0.0288117	0.000222503	0.168135	0.167775
rs7400002	0.0139701	0.000272443	0.230609	0.230135
rs74090351	-0.0250097	0.000650568	0.068143	0.069358
rs742036	-0.0143632	-0.000183678	0.375259	0.375093
rs7424120	-0.0123561	-0.000100558	0.601724	0.60103
rs75268115	-0.0207435	0.000359684	0.084699	0.0831571
rs75609851	-0.199116	0.000851994	0.01048	0.0104464
rs75942983	-0.0201303	-0.000277799	0.083627	0.0834039
rs7642634	-0.0165953	0.000230597	0.129958	0.130541
rs77009508	0.0450912	-0.000473787	0.073734	0.0751148
rs7704653	0.0157533	-0.000134558	0.723029	0.722962
rs7714361	0.0138858	0.000351766	0.233667	0.234084
rs7735249	0.0268635	0.000183097	0.112767	0.112718
rs7786102	-0.0283695	-2.11E-05	0.715703	0.718078
rs7786339	0.016328	7.75E-05	0.168936	0.168552
rs7847285	-0.0116104	0.000102129	0.589213	0.590115
rs78484485	-0.0758529	-0.000298194	0.054016	0.0534153
rs78588343	-0.0155231	0.000208124	0.176216	0.179157
rs7861679	0.0122025	-0.000120488	0.696815	0.696939
rs79153732	0.0773626	-0.000656255	0.017389	0.0168939
rs79192570	-0.0251844	-0.000126559	0.14416	0.143876
rs79287178	0.0500946	0.000163105	0.031151	0.0313199
rs79357714	-0.0289463	0.000108367	0.045724	0.0461369
rs7947951	0.0193777	0.00023867	0.689254	0.691404
rs80276949	0.0457563	0.000362646	0.022677	0.0231164
rs8102873	0.0123302	-2.74E-05	0.584919	0.584737
rs8126001	-0.0163778	0.000314787	0.489576	0.48972
rs852388	0.0157124	0.000258533	0.211278	0.211888
rs867939	-0.0136048	3.30E-05	0.57621	0.576725
rs880315	-0.011772	4.47E-05	0.340211	0.340623
rs921971	0.01565	0.000110703	0.265901	0.267167
rs9376511	-0.0154993	0.000116527	0.203288	0.202744
rs9425589	-0.0137618	-8.91E-05	0.566781	0.566393
rs9436661	-0.0777322	-0.000163561	0.352919	0.354907
rs9480889	0.0163067	0.00026949	0.783137	0.782706
rs954244	0.015323	5.24E-05	0.254641	0.253419
rs9561643	0.0167149	-4.83E-05	0.314726	0.313096
rs9584870	-0.0123562	-0.000261731	0.366179	0.366037
rs970069	0.0162322	0.000148038	0.212039	0.214623
rs9831084	-0.0118516	-0.000138336	0.461922	0.463138
rs9902027	-0.0151609	-8.74E-05	0.77437	0.774109
rs9908820	0.0123258	0.00024649	0.728481	0.732719
rs998584	0.0401182	0.000147407	0.482727	0.481711
