# Supplementary material for: Causal association between triglycerides and cholesterol-lowering medication with non-rheumatic valve disease: A 2-sample Mendelian randomization study
Source: Medicine (Baltimore). 2024 Jul 19;103(29):e38971. doi: 10.1097/MD.0000000000038971 (PMC11398802; doi:10.1097/MD.0000000000038971)
Supplement: Supplementary file 2 [file medi-103-e38971-s002.pdf]

SNP	beta.exposure	beta.outcome	eaf.exposure	eaf.outcome
rs1046801[illegible]	0.00828764	8.77E-05	0.296785	0.297034
rs1080433[illegible]	-0.00893165	-0.000104719	0.431132	0.431475
rs1081105	0.0295169	0.000382194	0.027535	0.0278866
rs1085714[illegible]	0.0080586	0.000232502	0.290189	0.291492
rs1105784[illegible]	0.0118059	0.000486751	0.141342	0.139493
rs1159114[illegible]	-0.072924	-0.000965346	0.017316	0.0176733
rs1169288	0.00874319	0.000167742	0.316017	0.313011
rs1175643[illegible]	0.0223122	0.000130349	0.031865	0.03216
rs1177333[illegible]	0.0459122	0.00126835	0.01838	0.0186119
rs1247313[illegible]	0.0100213	-0.000113112	0.257051	0.254457
rs1274037[illegible]	-0.0300486	-0.00033869	0.221547	0.221809
rs12916	0.0122803	-0.000143924	0.400541	0.399752
rs1292488[illegible]	0.0131321	8.10E-05	0.190627	0.188654
rs1414696[illegible]	0.0416551	0.000803523	0.009965	0.0101507
rs1421085	0.0085551	0.000154024	0.405858	0.401845
rs1477110[illegible]	0.0278194	-0.000345545	0.036352	0.0369754
rs1748275[illegible]	-0.016731	-0.000392259	0.100537	0.100762
rs1894400	0.00856163	0.000143152	0.326104	0.324989
rs1970112	0.0112784	0.000346411	0.476212	0.474813
rs2072633	0.00930064	3.06E-05	0.528113	0.522266
rs2207132	0.0311304	0.000394857	0.0329	0.0328715
rs2603192	-0.00725687	-0.000177155	0.415553	0.414003
rs2618567	-0.00776166	0.000200766	0.659419	0.660182
rs2738447	0.0129463	-0.000371018	0.59283	0.592668
rs2857861	0.00728057	0.000177656	0.53115	0.529206
rs2860176[illegible]	-0.0182783	-0.000233264	0.417405	0.419467
rs3446887[illegible]	0.0178478	-0.0001258	0.437987	0.441103
rs4299376	-0.0149868	-0.000225306	0.676663	0.676393
rs456598	0.010223	0.000188649	0.141055	0.142137
rs4646257	-0.00986619	0.000171395	0.81971	0.818498
rs4704727	0.00879317	-6.98E-07	0.659426	0.660055
rs5573049[illegible]	0.0339679	0.000711298	0.079684	0.0813848
rs5617956[illegible]	-0.00736949	3.29E-06	0.388009	0.389328
rs570530	0.00710617	0.000254721	0.504902	0.503199
rs5854292[illegible]	-0.0243947	-0.000740854	0.074756	0.0754304
rs6119470[illegible]	-0.0346613	6.70E-05	0.11904	0.118927
rs633185	0.0087548	2.51E-05	0.715446	0.715528
rs635634	0.0130329	-0.000229919	0.184492	0.184153
rs6780171	0.00842984	-0.000277942	0.31382	0.312771
rs693668	0.00902711	0.000152953	0.648423	0.650395
rs7412	-0.0483438	-8.59E-05	0.080309	0.0804374
rs7534572	0.0125699	0.000158569	0.647409	0.645627
rs7681302	-0.00897949	-0.000151086	0.80344	0.802151
rs7754216[illegible]	0.0338244	0.000149233	0.022534	0.0230084
rs7877715[illegible]	-0.0185279	-0.000266531	0.054615	0.0547763
rs7903146	0.0118356	-0.00010475	0.291607	0.290309
rs8126001	-0.00896638	0.000314787	0.489454	0.48972
rs964184	-0.0287774	-0.000129639	0.866923	0.867818
rs9647335	-0.0102426	0.000186522	0.192064	0.191563
